# (‐)‐Epigallocatechin‐3‐gallate induced apoptosis by dissociation of c‐FLIP/Ku70 complex in gastric cancer cells

**DOI:** 10.1111/jcmm.17873

**Published:** 2023-08-03

**Authors:** Mahtab Shahriari Felordi, Mehdi Alikhani, Zahra Farzaneh, Mahmoud Alipour Choshali, Marzieh Ebrahimi, Hamidreza Aboulkheyr Es, Abbas Piryaei, Mustapha Najimi, Massoud Vosough

**Affiliations:** ^1^ Department of Regenerative Medicine, Cell Science Research Center Royan Institute for Stem Cell Biology and Technology, ACECR Tehran Iran; ^2^ Department of Stem Cells and Developmental Biology, Cell Science Research Center Royan Institute for Stem Cell Biology and Technology, ACECR Tehran Iran; ^3^ School of Biomedical Engineering University of Technology Sydney Sydney New South Wales Australia; ^4^ Department of Biology and Anatomical Sciences, School of Medicine Shahid Beheshti University of Medical Sciences Tehran Iran; ^5^ Department of Tissue Engineering and Applied Cell Sciences, School of Advanced Technologies in Medicine Shahid Beheshti University of Medical Sciences Tehran Iran; ^6^ Laboratory of Pediatric Hepatology and Cell Therapy Institute of Experimental and Clinical Research (IREC) Brussels Belgium; ^7^ Experimental Cancer Medicine, Department of Laboratory Medicine Karolinska Institute Stockholm Sweden

**Keywords:** apoptosis induction, c‐FLIP/Ku70 complex, EGCG treatment, epigenetic modification, gastric cancer, histone deacetylation

## Abstract

Anti‐cancer properties of (‐)‐epigallocatechin‐3‐gallate (EGCG) are mediated via apoptosis induction, as well as inhibition of cell proliferation and histone deacetylase. Accumulation of stabilized cellular FLICE‐inhibitory protein (c‐FLIP)/Ku70 complex in the cytoplasm inhibits apoptosis through interruption of extrinsic apoptosis pathway. In this study, we evaluated the anti‐cancer role of EGCG in gastric cancer (GC) cells through dissociation of c‐FLIP/Ku70 complex. MKN‐45 cells were treated with EGCG or its antagonist MG149 for 24 h. Apoptosis was evaluated by flow cytometry and quantitative RT‐PCR. Protein expression of c‐FLIP and Ku70 was analysed using western blot and immunofluorescence. Dissociation of c‐FLIP/Ku70 complex as well as Ku70 translocation were studied by sub‐cellular fractionation and co‐immunoprecipitation. EGCG induced apoptosis in MKN‐45 cells with substantial up‐regulation of *P53* and *P21*, down‐regulation of *c‐Myc* and *Cyclin D1* as well as cell cycle arrest in S and G2/M check points. Moreover, EGCG treatment suppressed the expression of c‐FLIP and Ku70, decreased their interaction while increasing the Ku70 nuclear content. By dissociating the c‐FLIP/Ku70 complex, EGCG could be an alternative component to the conventional HDAC inhibitors in order to induce apoptosis in GC cells. Thus, its combination with other cancer therapy protocols could result in a better therapeutic outcome.

## 1 INTRODUCTION

Gastric cancer (GC) is the fifth most common cancer with the fourth highest mortality rate in the world.^1^ GC patients often present several symptoms, including dyspepsia and reflux. Advanced stages of GC are usually characterized by progressive signs such as dysphagia, loss of weight, gastrointestinal bleeding and anaemia.^2^ Surgery combined with chemotherapy is still the only available treatment method for non‐metastatic GC. Despite the advances in early diagnosis and chemotherapy protocols, current therapeutic approaches for GC have still low efficacy due to multidrug resistance.^3^, ^4^

Preventive methods aimed at limiting the risk factors and accelerating early detection of precancerous lesions.^5^ Accordingly, discovering new treatment strategies is an essential and remarkable priority.^6^, ^7^ For instance, novel classification of GC based on different molecular subtypes and specific biomarkers resulted in a more precise personalized therapy.^2^ Understanding tumour biology will also lead to the development of effective targeted therapies based on the molecular aetiology of cancer.^8^, ^9^ Chemoprevention for GC using natural, synthetic or biological agents is another therapeutic approach under evaluation.^10^, ^11^ Combined anticancer therapy based on molecular targets and using approved natural components could be an innovative strategy to induce anti‐apoptotic pathways.^12^

In many solid tumours and hematologic malignancies, abnormalities in histone acetylation and/or histone deacetylases (HDACs) may contribute to carcinogenesis.^13^ The overexpression of HDACs induces tumour cell growth and resulted in poor prognosis for various cancers.^14^, ^15^ Also, HDACs are implicated in the proliferation, apoptosis, invasion and migration of a variety of cancer cells, including GC cells.^16^ Therefore, HDACs were considered as potential targets in the therapeutic approaches developed for different cancers.^17^ Although HDACs have the leading role in the deacetylation of histones, they also play an important role in the deacetylation of multiple non‐histone proteins and modulation of their activity, cell localisation of proteins and protein–protein interactions.^17^ Ku70 is one of the non‐histone proteins which is targeted by HDACs. This protein evolved in tumorigenesis through regulating cell death.^18^ Ku70 is a nuclear DNA repair factor that forms a heterodimer with Ku80 and binds to double‐strand DNA (dsDNA). Ku70/Ku80 dimer formation is necessary for DNA repair via the classical non‐homologous end‐joining (c‐NHEJ) pathway.^19^, ^20^ In cancer cells, Ku70 can translocate from nucleus to the cytoplasm because of the increased levels of HDACs.^21^ The function of cytosolic deacetylated Ku70 as a cell death regulator has already been investigated because of its association with apoptotic proteins.^22^ One of the critical anti‐apoptotic proteins in cancer cells is cellular FLICE‐inhibitory protein (c‐FLIP). This protein could be a potential anti‐cancer target in order to induce apoptosis via blocking caspase 8 activation and binding to the FAS‐associated death domain (FADD).^4^, ^23^ c‐FLIP and Ku70 interact and bond together in the cytoplasm of cancer cells. Ku70 stabilizes c‐FLIP protein via its C‐terminal region and prevents its degradation in the cytoplasm. Furthermore, acetylation of Ku70 inhibits its interaction with c‐FLIP and results in c‐FLIP degradation by the ubiquitin proteasome system.^4^, ^24^ Thus, HDAC inhibition induces dissociation of c‐FLIP/Ku70 complex after acetylation of cytosolic Ku70. This dissociation restores apoptosis through the extrinsic pathway by decreasing cytosolic c‐FLIP levels.^17^ Therefore, novel therapies have focused on HDAC proteins as potential targets and an alternative approach in cancer treatment by using various components such as histone deacetylases inhibitors (HDACi).

Suberoylanilide hydroxamic acid (SAHA) was FDA approved in 2006 and is one of the effective HDACi for the treatment of cutaneous T‐cell lymphoma (CTCL).^25^ SAHA disrupts any complex between Ku70 and other proteins through Ku70 acetylation.^4^ In addition, several studies showed that this dissociation causes cell death through induction of extrinsic apoptosis pathway by decreasing the level of c‐FLIP in cancer cells.^4^, ^26^ Although SAHA is a potential HDACi, the use of natural components with the same mechanisms of action could be more convenient and probably with less side effects in cancer.

(‐)‐epigallocatechin‐3‐gallate (EGCG), which comprises more than 50% of total polyphenols in green tea,^27^ has numerous potential chemo‐preventive and therapeutic functions for various tumours.^28^, ^29^ EGCG is a natural HDACi that can induce apoptosis via epigenetic modification as demonstrated in cancerous cells.^30^ Thus, it could be a potential natural HDACi that may be applied for cancer treatment. In addition, some studies showed that EGCG induces apoptosis in cancer cells through acetylation of Ku70 and can cause dissociation of the Bax/Ku70 complex as shown in lung cancer.^31^, ^32^ However, there is no clear evidence yet that EGCG treatment induces apoptosis through the dissociation of c‐FLIP/Ku70 complex.

The present study aimed to investigate the effect of EGCG on apoptosis induction and check its possible molecular mechanism on GC cells. Dissociation of c‐FLIP/Ku70 complex in GC cells after EGCG treatment could induce a programmed cell death.

## 2 MATERIALS AND METHODS

### 2.1 Reagents

EGCG (458.4 Da, ≥ 98% purity, ab120716) and MG149 (Cat. #. 22135), a small molecule inhibiting histone acetyltransferase (HATi) and acting as an antagonist for HDACi,^33^ were purchased from Abcam's and Cayman Chemical Company's representatives respectively. EGCG was dissolved in double‐deionized filter‐sterilized water; MG149 was dissolved in dimethyl sulfoxide (DMSO) (Sigma‐Aldrich Cat. # 67685), and both of them were stored at −20°C. Other reagents which were used in this study are listed as follows: Roswell Park Memorial Institute‐1640 medium (RPMI1640, Cat.# 1880348), non‐essential amino acids (NEAA, Cat.# 11140035), L‐glutamine (Cat.# 35050061), 0.05% Trypsin–EDTA (1X, Cat.# 25300054) and foetal bovine serum (FBS, Cat.# 10270106) from Gibco®, Life Technologies™, Paisley, UK; human Platelet Lysate (hPL) from CellTech Pharmed company (CTP, 10321); penicillin–streptomycin (P/S) from Thermo Fischer Scientific (Cat.# 15070083); rabbit monoclonal anti‐Ku70 primary antibody (Abcam, Cat.# ab92450); mouse monoclonal anti‐c‐FLIP S/L primary antibody (Santa Cruz Biotechnology, Cat.# G‐11_sc‐5276); HRP‐conjugated secondary antibodies (Sigma‐Aldrich, A2554 anti‐mouse and A0545 anti‐rabbit); donkey anti‐mouse IgG Alexa Fluor®488 (Invitrogen, A‐21202); donkey anti‐rabbit IgG Alexa Fluor®546 (Invitrogen, A10040). DAPI (Invitrogen, D1306); Phosphatidyl Serine‐ FITC kit (IQ product, Groningen, Netherlands (IQP‐116F)), MN mRNA extraction Kit (Ref: 740955.10, MACHEREY‐NAGEL, GmbH & Co.); complementary DNA (cDNA) synthesis kit (Ref: A101161, Pars Tous biotechnology kit); Taq DNA Polymerase Master Mix (Takara Bio, Inc., SYBR Premix Ex Taq II RR820A; RIPA buffer (Sigma‐Aldrich. MFCD02100484); protease inhibitor (Sigma‐Aldrich, P2714); phosphatase inhibitor (Sigma‐Aldrich. P2850); BCA assay (Thermo Fisher Scientific); nonfat dry milk (Cell Signaling Technology, #9999S) from; ECL western blotting detection reagent (Thermo Scientific, RPN2232); 0.1% NP‐40 (Sigma‐Aldrich, #74388), and Co‐IP Kit (Sigma‐Aldrich. IP50).

### 2.2 Cell culture

The study and related experiments were all approved by the competent ethical committee (IRC98‐0335). MKN‐45 cells were obtained from Royan Institute Cell Bank (Tehran, Iran), and cultured in RPMI 1640 supplemented with 5% hPL, 1% NEAA, 1% L‐glutamine, 100 U/mL penicillin, and 100 U/mL streptomycin in a humidified 5% CO_2_ incubator at 37°C. The medium was changed every day. Twenty‐four hours after seeding, the cell culture media were replaced by medium supplemented with 5% hPL. In all assays, the cells were incubated with appropriate concentrations of EGCG and MG149 for 24 h.

### 2.3 Evaluation of the effective Dose

The effect of EGCG and MG149 on apoptosis rate of MKN‐45 cells was analysed using Annexin V/PI double staining analyses. Cancerous cells were plated at a density of 2 × 10^5^ cells/well in a 6‐well plate. On the following day, the cells were incubated with hPL‐free media before treatment with EGCG (100 μM) and MG149 (0.1, 1, 5, 20 and 100 μM) for 24 h (Figure S1).

### 2.4 Apoptosis analysis

MKN‐45 cells were seeded in 6‐well tissue culture plates (2 × 10^5^ cells/well). After starvation and treatment with 100 μM EGCG and 1 μM MG149 for 24 h, the floating cells in the medium were collected. The adherent cells were detached using 0.05% trypsin–EDTA. Then, the fresh culture medium containing hPL was used to inactivate the enzyme. After gently homogenising the suspension, the cells were centrifuged for 5 min at 500 rpm. The cells were stained with Annexin V‐FITC and PI according to the manufacturer's instructions. Untreated cells were used as controls. The number of dead cells was analysed by FACSCalibur (BD, Franklin Lakes). The data were analysed using FlowJo 9.0 software (Tree Star).

### 2.5 Cell cycle analysis

MKN‐45 cells were plated at a density of 5 × 10^5^ cells/well in 6‐well tissue culture plates. After treatment, the cell cycle profile was assayed by flow cytometry after staining with PI/RNase. Cells were fixed gently with cold ethanol (80%) and kept at 4°C overnight. Then, the cells were resuspended and gently dissociated by pipetting in 3 mL of PBS and centrifuged at 4°C. Finally, 100 μL RNase were added, and after 20 min of incubation at 37°C, cell cycle analysis was performed using FACSCalibur (BD) and FlowJo 9.0 software (Tree Star).

### 2.6 Quantitative real time‐PCR assay

After seeding 8 × 10^5^ cells/well (6‐well plate) and treatment with 100 μM EGCG and 1 μM MG149, total mRNA was extracted from each group according to the manufacturer's protocol. RNA concentrations were measured at 260 nm, and the mRNA purity was spectrophotometrically evaluated at 260 and 280 nm. Two micrograms of RNA was used as a template for complementary DNA (cDNA) synthesis. The primer sequences used in the study are listed in Table 1. Real‐time PCR was performed with Taq DNA Polymerase Master Mix and the StepOnePlus™ Real‐Time PCR System. Data were analysed and presented using the comparative CT method (2^−ΔΔCt^).

**TABLE 1:** Sequences of primers used in this study.

Gene	Forward (5′–3′)	Reverse (5′–3′)
*p53*	CCAC CATCCACTACAACTAC	AAACACGC ACCTCAAAGC
*p21*	CTGGAGACTCTCAGGGTCGAA	CGGCGTTTGGAGTGGTAGAA
c‐Myc	GTCAAGAGGCGAACACACAAC	TTGGACGGACAGGATGTATGC
*Cyclin D1*	GCTGCGAAGTGGAAACCATC	CCTCCTTCTGCACACATTTGAA
*GAPDH*	GACTTCAACAGCAACTCCCAC	TCCACCACCCTGTTGCTGTA

### 2.7 Immunofluorescence staining

MKN‐45 cells were plated (75 × 10^4^ cells/well) and treated with 100 μM EGCG and 1 μM MG149. The MKN‐45 cells present specific characteristics during in vitro culture as a subpopulation of cells detach from the surface of the plate and make aggregates after seeding. In our study, both adherent and aggregating MKN‐45 cells from each culture condition were analysed. After collection and pooling, corresponding cell pellets were fixed in 4% paraformaldehyde for 20 min at room temperature. Thereafter, the fixed cells were harvested and suspended in compact 1% agarose, and after jellification, the agarose blocks were subsequently embedded in paraffin for further use. The paraffin blocks were sectioned using microtome into 5 μm slices, then deparaffinized and stained with haematoxylin and eosin. Subsequently, sections were rehydrated using standard procedures. Antigens were retrieved in boiling citrate buffer (10 mM citric acid, 0.05% Tween 20, pH 6.0) for 20 min. The sections were thereafter permeabilized with 0.5 or 0.3% Triton‐X100 for Ku70 and c‐FLIP, respectively, and incubated with 1% BSA (A9418, Sigma‐Aldrich) for 1 h to block non‐specific antibody binding sites. After washing with PBS‐Tween (PBS with 0.05% of Tween 20) three times for 15 min, the sections were incubated overnight with primary anti‐mouse (at 1:20) or anti‐rabbit (at 1:200) antibodies at room temperature. For negative controls, PBS was used instead of the primary antibody. Sections were washed three times for 5 min in PBS‐Tween, and thereafter incubated for 1 h at RT with corresponding secondary antibodies (at 1:500). Finally, the sections were incubated with DAPI to label nuclei for 1 min at room temperature, after that washed in PBS‐Tween three times for 5 min. The sections were examined using an Olympus BX51 microscope (Olympus). To quantify the percentage of positive cells in each group, ImageJ software was used according to the instructor's protocol. We checked at least six images with the same magnification (scale bar = 100 μm) from different sections in each group.

### 2.8 Western blot analysis

Cultured cells were plated at 8 × 10^5^ cells/well density (6‐well plate). After treatment with 100 μM EGCG and 1 μM MG149, cells were lysed in RIPA buffer containing protease and phosphatase inhibitors. Sonication was used with the frequency of 60 kHz, three pulses for the 30 s, and then the cell lysate centrifuged (16,000 × ***g*** at 4°C) for 20 min. Protein concentration in the supernatant was determined by the BCA assay kit, and sample equalized aliquots were mixed with the SDS sample buffer and loaded onto a gel after boiling. Protein extracts containing 30 μg of total proteins were resolved by 12% polyacrylamide gel electrophoresis, transferred onto polyvinylidene difluoride (PVDF) membranes, and blocked using nonfat dry milk in TBST (10 mM Tris–HCl pH 7.6, 150 mM NaCl, 0.1% Tween 20) for 1 h at RT. The membrane was then incubated with primary antibodies for Ku70 (at 1:5000) in TBST for 2 h and anti c‐FLIP (at 1:1000) in TBST overnight at 4°C. Then, the membranes were incubated with HRP‐conjugated secondary antibodies (at 1:100000) in a 1% blocking buffer for 1 h. The membranes were washed three times with TBS‐Tween after each step. The signals were detected using Thermo Scientific Super Signal West Femto Maximum Sensitivity Substrate or Amersham ECL western blotting detection reagent to visualize the bands normalized to the corresponding GAPDH levels. The blot exposures were measured using Uvitec (Alliance Q9 Advanced) equipment.

### 2.9 Sub‐cellular fractionation technique

Cytoplasm and nuclei of the treated cells were separated, as described in Nabbi and Riabowol's protocol.^34^ Briefly, MKN‐45 cells were scraped using PBS containing protease inhibitors, suspended and centrifuged for 10 s at 10,000 rpm. The cell pellet was resuspended in ice‐cold PBS^−^ containing 0.1% NP‐40 (as whole protein). Then, the remaining lysed cells were centrifuged for 30 s at 10 K rpm. The supernatant was separated as the cytoplasmic fraction. The pellet was resuspended in PBS^−^ containing 0.1% NP‐40 and centrifuged again for 30 s at 10,000 rpm. The pellet was resuspended with a 1× Laemmli sample buffer (nuclear fraction). The whole‐cell proteins and nuclear fraction were sonicated with the 20 kHz frequency, two pulses for 16 s. Finally, to quantify the target proteins, immunoblotting assay was used.

### 2.10 Co‐immunoprecipitation assay

To assess c‐FLIP/Ku70 and the effect of EGCG treatment on this complex, co‐immunoprecipitation (Co‐IP) assay was performed using Co‐IP Kit (Sigma). The treated cells were lysed in Co‐IP buffer including 20 mM Tris–HCl, pH 8.0; 1% Nonidet P‐40; 10% glycerol; 1 mM MgCl2; 1 mM EDTA; 150 mM NaCl and protease and phosphatase inhibitors. Then, the cell lysate proteins were incubated overnight with the Ku70 primary antibody. Subsequently, 50 μL of protein‐G Sepharose beads were washed with lysis buffer, added to the solution and incubated for 2 h at 4°C in a rocker mixer. The mixture was then subjected to the purification column and centrifuged at 10000 g for 1 min and washed five times with washing buffer. Finally, 50 μL of 1X Laemmli sample buffer was added, mixed evenly, boiled at 95°C for 7 min and centrifuged. The eluates were analysed by immunoblotting.

### 2.11 Statistical analyses

Data were presented as mean ± SD. Statistical analyses were performed using GraphPad Prism 8.4.3 (GraphPad). Differences between groups were compared using anova and *t*‐test followed by LSD test. The *p* < 0.05 was considered as statistically significant (*n* = 3).

## 3 RESULTS

### 3.1 EGCG treatment induces apoptosis and upregulates the expression of related genes in MKN‐45 cells

**FIGURE 1:**
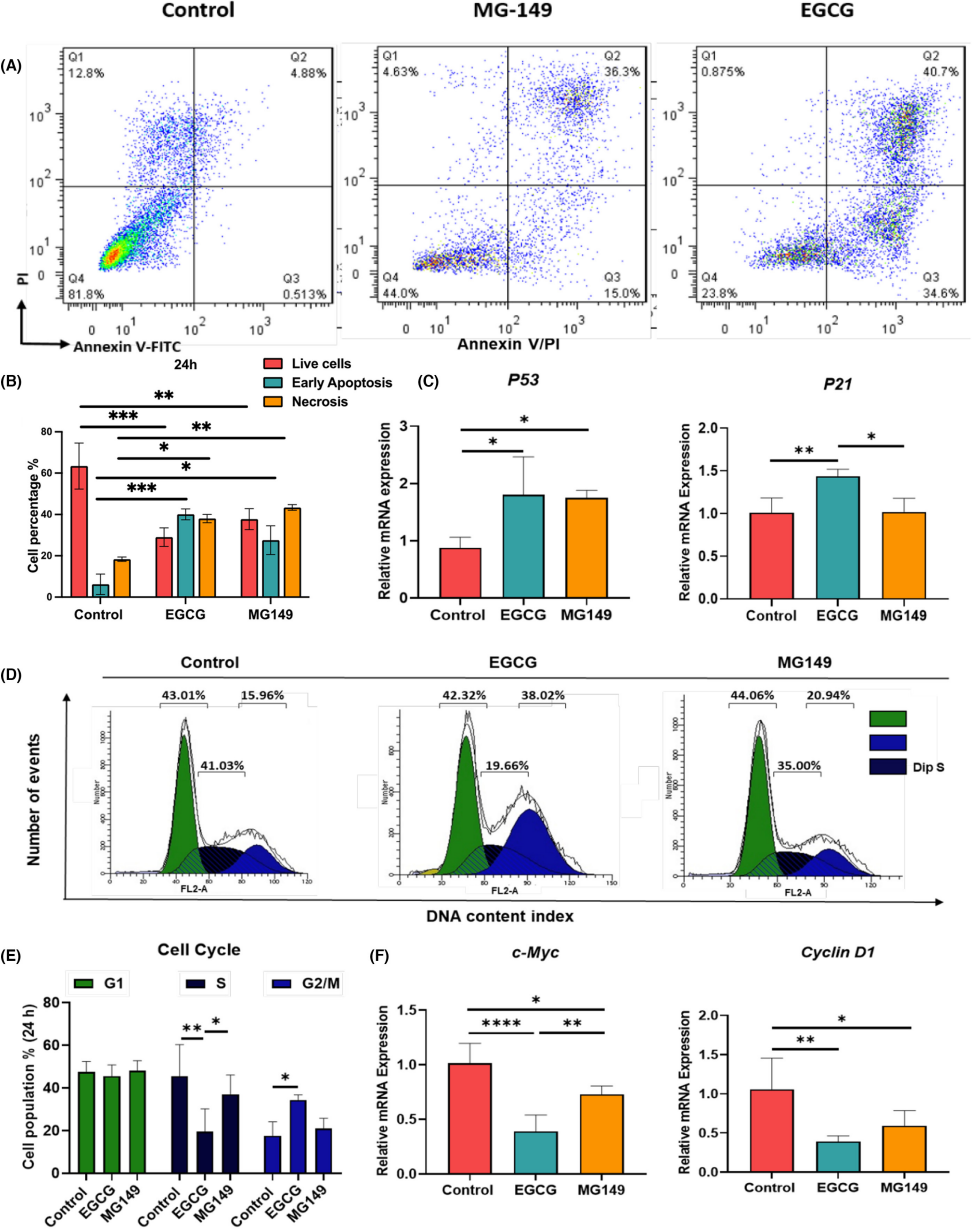
Analysis of cell cycle and apoptosis induction after EGCG treatment. (A, B) EGCG induces dose‐dependent apoptosis in MKN‐45 cells at the early apoptosis phase. Cells were treated with EGCG and MG149 for 24 h. Apoptotic cells were stained with Annexin V/PI kit and determined by flow cytometry analysis. The cells were analysed using Graph Pad Prism‐8 software. Data are shown as mean ± SD (*n* = 3). Statistical analysis was performed using one‐way anova (* *p* < 0.05, ** *p* < 0.01, *** *p* < 0.001 and **** *p* < 0.0001). (C) *P53* and *P21* gene expression in EGCG and MG149 treated groups was compared to the non‐treated control group. Data were normalized to *GAPDH* and were shown as mean ± SD (*n* = 3). Statistical analysis was performed using unpaired two‐tailed Student's *t*‐tests. * *p* < 0.05 and ** *p* < 0.01. (D, E) Flow cytometry analysis of cell cycle progression after EGCG and MG149 treatment and its regulatory mechanism. Cell cycle assays revealed that EGCG treatment significantly increased the ratio of cells in the G2 phase and decreased the ratio of cells in the S phase after 24 h (*n* = 5). (F) The expression of *c‐Myc* and *Cyclin D1* genes after treatment with EGCG and MG149 was compared to the non‐treated control group. Data were normalized to *GAPDH* and are shown as mean ± SD (*n* ≥ 3).

Several studies have proposed 100 μM EGCG could be an optimal concentration to treat cancer cells.^35^, ^36^ Accordingly, we incubated MKN‐45 cells with this EGCG concentration and evaluated its potential to induce cell apoptosis by using flow cytometry. Our data revealed that the number of live cells in EGCG treated group was significantly lower (29 ± 4%) as compared to the non‐treated control group (62 ± 11%) (*p* < 0.001). Indeed, most of the cells in the EGCG group were in the early apoptosis and necrosis phases. The percentage of MKN‐45 cells undergoing early apoptosis (Annexin V^+^/PI^+^) was increased significantly by almost seven times (38 ± 3%) after 24 h treatment with 100 μm EGCG (*p* < 0.001) as compared to non‐treated cells (5 ± 3%) (Figure 1A,B). On the one hand, the number of cells in necrosis phase (Annexin V^+^/PI^−^) seemed also increased (35 ± 4%) as compared to non‐treated cells (18 ± 6%) (p < 0.05). The number of live cells following the treatment with MG149 was decreased compared to the non‐treated control group (*p* < 0.01) (Figure 1A,B). On the other hand, the number of cells in early apoptosis (27 ± 9%) (*p* < 0.05) and necrosis phases had increased in this group compared to the control group (*p* < 0.01) (Figure 1A,B).

In addition, we did analyse the mRNA expression of *P53* and *P21* by using quantitative RT‐ PCR after treatment with 100 μm EGCG and 1 μm MG149 (*n* = 3). Our results showed that *P53* (*p* < 0.05) and *P21* (*p* < 0.01) mRNAs were significantly upregulated (2 times and 1.5 times respectively) in EGCG‐treated cells as compared to the non‐treated control group (Figure 1C). when treated with 1 μm MG149, only the expression level of *P53* was significantly increased (two times) as compared to the control group (*p* < 0.05), while no significant effect was measured for *P21* mRNA expression (Figure 1C).

### 3.2 EGCG treatment induces G2/M cell cycle arrest and decreases the number of cells in S phase

Our data demonstrated that EGCG treatment of MKN‐45 cells induced an ~38% increase of cells in G2/M phase (*p* < 0.05), while the number of cells in S phase decreased by ~19% as compared to the control non treated cell group (*p* < 0.01) (Figure 1D,E). The expression of *c‐Myc* and *Cyclin D1*, two crucial genes involved in the S and G2/M phases, was also quantified. After 24 h treatment with 100 μm EGCG, the mRNA expression of *c‐Myc* and *Cyclin D1* was significantly decreased up to 50% for both genes as compared to the control non‐treated cells (*c‐Myc* [*p* < 0.0001] and *Cyclin D1* [*p* < 0.01]) (Figure 1F). These results indicated that 100 μm EGCG treatment could induce apoptosis in MKN‐45 cells, which can cause G2/M arrest in these cells. No correlation was found with the number of cells in G1 phase. When GC cells were treated with MG149, the mRNA expression of *c‐Myc* and *Cyclin D1* was significantly decreased compared to the non‐treated control group (*c‐Myc* (*p* < 0.05) and *Cyclin D1* (*p* < 0.05) (Figure 1F).

### 3.3 EGCG treatment decreases cytoplasmic c‐FLIP and increases the nuclear localisation of Ku70 in MKN‐45 cells

**FIGURE 2:**
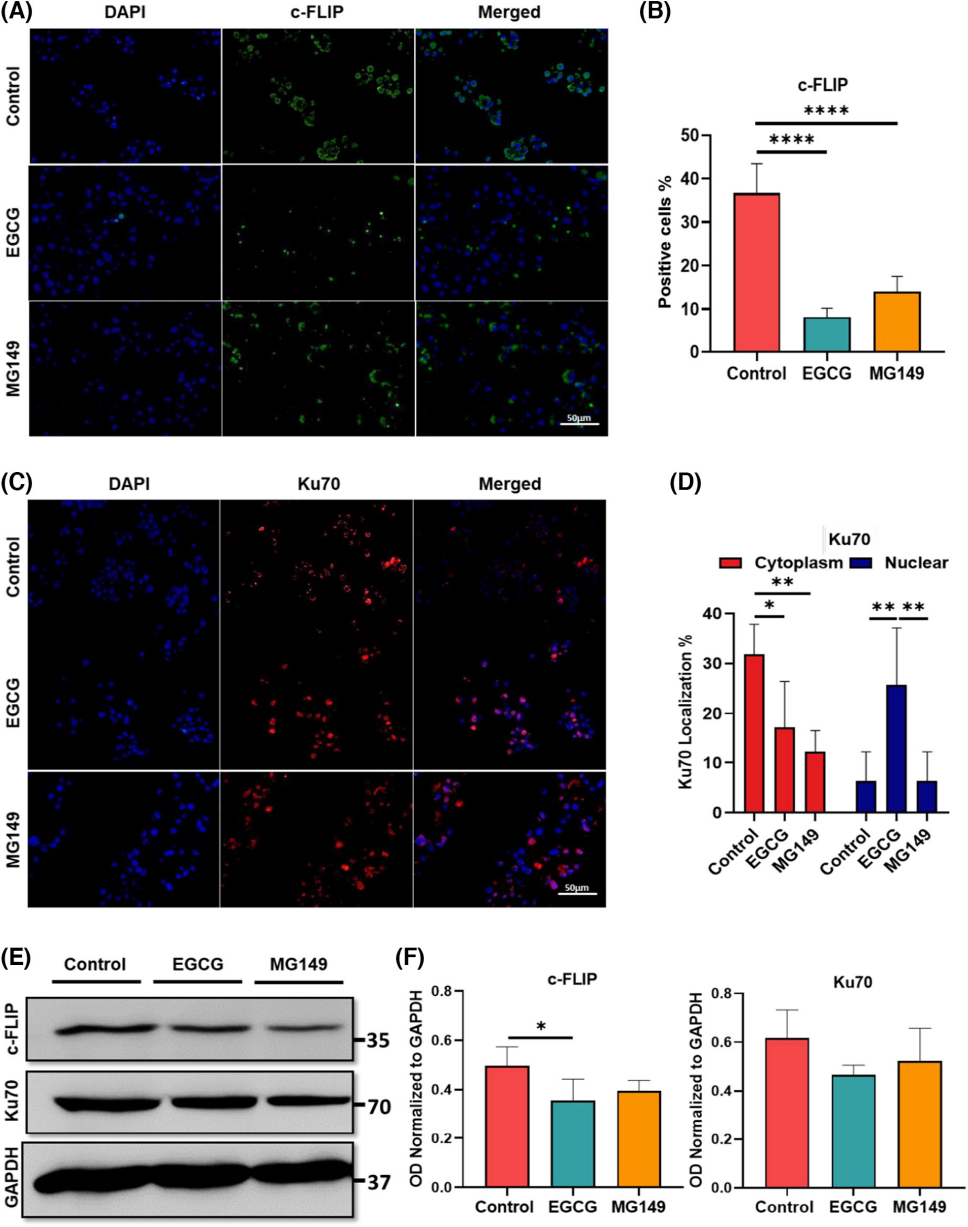
The expression and localisation analysis of c‐FLIP and Ku70 before and after treatment with EGCG. (A, B) The expression level of c‐FLIP after treatment with EGCG was significantly decreased. (C, D) Ku70 protein after treatment with EGCG was translocated from cytoplasm to the nucleus and the level of cytoplasmic Ku70 was declined. (E, F) Protein expression of c‐FLIP and Ku70 following treatment with EGCG after 24 h using western blot. The semi‐quantitative results demonstrated that EGCG treatment significantly decreased the expression of c‐FLIP compared to control group while MG149 did not induce any changes compared to the non‐treated control group. Scale bar: 50 μm.

c‐FLIP and Ku70 interact in the cytoplasm of cancer cells. Ku70 stabilizes c‐FLIP protein via its C‐terminal region in the cytoplasm thereby preventing its degradation.^4^, ^24^ We evaluated the number of c‐FLIP immune‐positive cells and the subcellular localisation of Ku70 before and after EGCG treatment for 24 h, using immunofluorescence on fixed cells. As shown in Figure 2A,C, the number of c‐FLIP positive cells was significantly reduced (almost 25%) after treatment with EGCG (*p* < 0.0001) compared to the non‐treated control group (Figure 2A,B). Also, Ku70 immunostaining revealed its translocation (almost 15% decrease in cytoplasm and 20% increase in the nucleus) from cytoplasm to the nucleus after EGCG treatment (Figure 2C,D) *(p* < 0.05 and *p* < 0.01 for cytoplasm and nucleus respectively). When MKN‐45 cells were treated with MG149, the number of c‐FLIP positive cells was significantly reduced (almost 20%) *p* < 0.0001 (Figure 2A,B). The Ku70 content after treatment with MG149 was inversely decreased in the cytoplasm of GC cells (*p* < 0.01). The number of Ku70 positive cells in MG149 treated group did not show any difference as compared to the non‐treated control group.

We also assessed the protein expression of c‐FLIP and Ku70 following 24 h treatment with EGCG by using western blot test. The semi‐quantitative results demonstrated that EGCG treatment significantly decreased the expression of c‐FLIP while MG149 did not cause any changes compared to the non‐treated control group. Our data also reveal no changes in the protein expression level of Ku70 when MKN‐45 cells were treated with EGCG or MG149 (Figure 2E,F).

### 3.4 EGCG reduced cytoplasmic localisation c‐FLIP and induced nuclear translocation of Ku70

**FIGURE 3:**
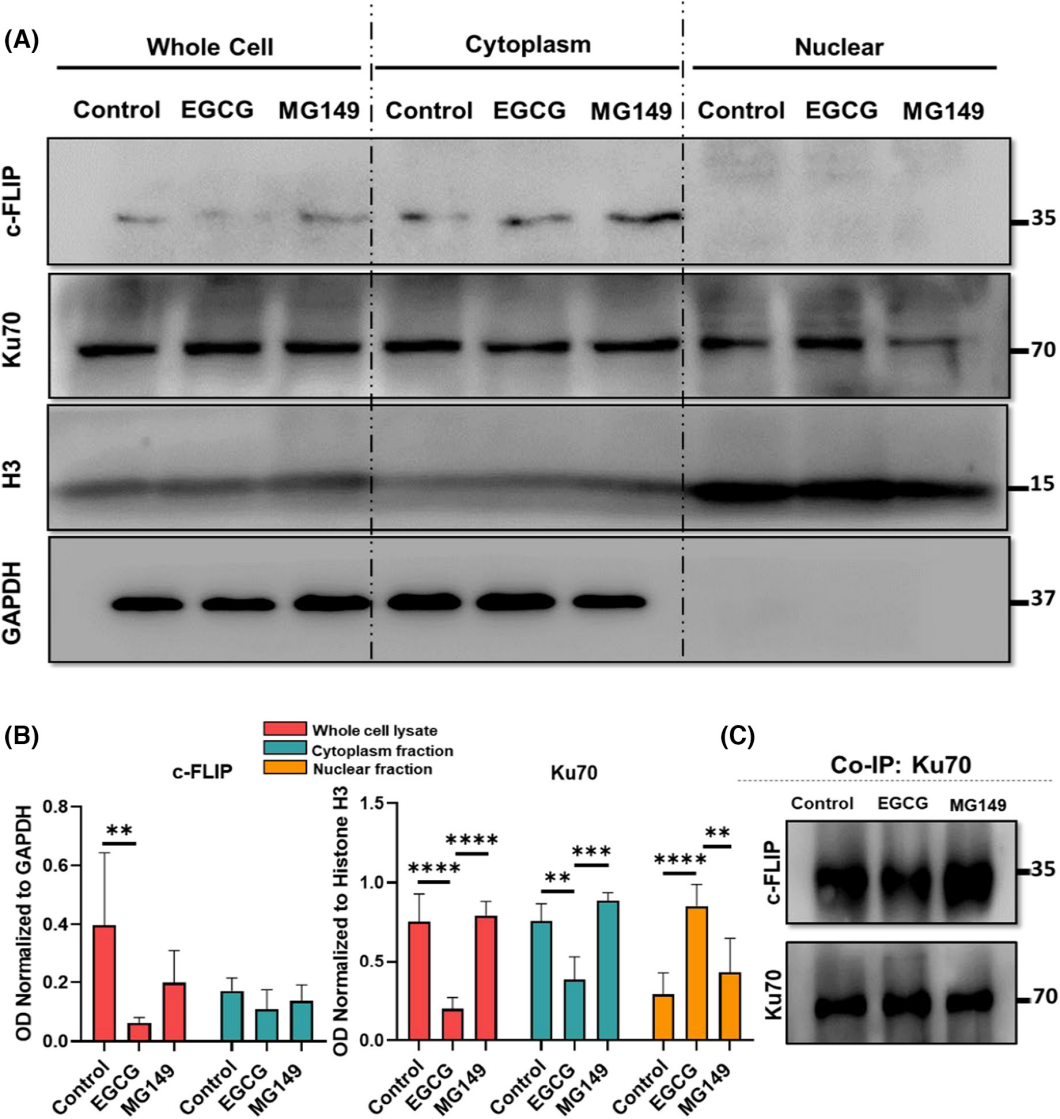
Evaluation of c‐FLIP/Ku70 complex localization. (A, B) Evaluation of c‐FLIP and Ku70 localization before and after treatment with 100 μM EGCG by the subcellular fractionation method. The expression level of c‐FLIP and Ku70 was normalized with their corresponding markers. GAPDH was used as a cytoplasmic control marker, and histone H3 as a nucleus control marker. (C) The interaction between c‐FLIP and Ku70 was evaluated before and after treatment with EGCG by Co‐IP assay. After treatment with EGCG, we observed that the c‐FLIP/Ku70 complex was decreased.

To explore the effect of EGCG treatment on the c‐FLIP level in the cytoplasm and the level of Ku70 in both cytoplasm and nucleus, the subcellular fractionation analysis was performed. First, the pools of cytoplasmic and nucleus proteins were extracted and c‐FLIP and Ku70 protein levels assessed in each fraction. As shown in Figure 3A,B, the total c‐FLIP protein level in whole‐cell lysate was significantly decreased (*p* < 0.01) after treatment with EGCG. Also, the Ku70 content in the nuclear fraction of EGCG‐treated cells was increased (Whole cell lysate [*p* < 0.0001], cytoplasmic [*p* < 0.01] and nuclear [*p* < 0.0001]) fractions (Figure 3B). In the MG149‐treated group, we did not notice any significant change in the c‐FLIP content after treatment compared to the non‐treated control group (Figure 3B).

### 3.5 EGCG treatment dissociates the c‐FLIP/Ku70 complex in GC cells

Following treatment of MKN‐45 cells with 100 μm EGCG for 24 h, co‐immunoprecipitation assay was used to detect the interaction between c‐FLIP/Ku70 in the cytoplasm. The results showed that after EGCG treatment, Ku70 translocated from cytoplasm to the nucleus and the level of Ku70 in cytoplasm was reduced. This could have been happened due to the dissociation of c‐FLIP/Ku70 complex (Figure 3C).

## 4 DISCUSSION

Recently, development of components inducing apoptosis, especially natural components, has gained remarkable interest in cancer therapy.^37^, ^38^ In our study, treatment of the MKN‐45 cells by EGCG, a natural component comprising more than 50% of total polyphenols in green tea,^27^ significantly induced their apoptosis as demonstrated by flow cytometry and Annexin V/PI staining analysis. In parallel, EGCG treated GC cells display substantial up‐regulation of *P53* and *P21*, and downregulation of *c‐Myc* and *Cyclin D1* which were associated with cell cycle arrest in S and G2/M check points. EGCG treatment of MKN‐45 cells also suppressed the expression of c‐FLIP and Ku70 proteins and significantly decrease their interaction as shown by co‐immunoprecipitation. As consequence, EGCG treatment has led to an increased nuclear translocation of Ku70 as shown by cell fractionation analyses. Huang and colleagues showed that that after trypsinization, the proteins that regulate cell metabolism, growth regulation, mitochondrial electron transportation and cell adhesion were downregulated and proteins that regulate cell apoptosis were upregulated. Further study showed that bcl‐2 was downregulated, p53 and p21 were both upregulated after trypsinization.^39^ In another study, Ni Yan et al. showed that trypsin induced apoptosis. However, this ratio was within the acceptable range.^40^

c‐FLIP, a master anti‐apoptotic factor, was shown to inhibit apoptosis in cancer cells through its binding to Ku70^41^, ^42^ (Figure 3C). Ku70/Bax complex is an anti‐apoptotic complexes and after Ku70 acetylation, this complex dissociates. Dissociation of this Bax/Ku70 complex, enables Bax to translocate into the mitochondria and induce apoptosis by caspase activation.^4^, ^31^ Moreover, regarding the extrinsic pathway, c‐FLIP is an essential anti‐apoptotic protein which inhibits the activation of apoptosis. This inhibition mediated by death receptors and it blocks activation of procaspase 8 via complexes termed DISCs (death‐inducing signalling complexes).^4^ Several studies showed that binding different proteins to Ku70 through some specific binding sites stabilizes cytoplasmic Bax/Ku70 complex and inhibits apoptosis in the cancer cells.

Zou et al. showed that binding of caveolin‐1 to Ku70 inhibits in colon cancer cells. According to our results, based on the HDAC inhibitory effect of EGCG, we proofed that after treatment with EGCG and because of deacetylation of Ku70, the c‐FLIP/Ku70 complex dissociated.^43^ In several studies, accumulation of c‐FLIP/Ku70 complex was indicated as an important phenomenon to inhibit apoptosis. The extra amounts of this complex were detected in different cancer cells like HepG2 cells, in which acetylation of Ku70 and break‐up of c‐FLIP/Ku70 complex facilitated ubiquitination of c‐FLIP and resulted in more vulnerability of the cancerous cells to apoptosis through increasing TNF‐related apoptosis‐inducing ligand (TRAIL) as crucial apoptosis primarily factor.^44^ In another study, treatment with two compounds, 10d and 10e, two analogues of cyano‐3‐oxo‐1,9‐dien glycyrrhetinic acid, induced apoptosis in HL‐60 cells and its association downregulated expression of c‐Flip.^41^ In addition, Tao et al. reported that acetylation of Ku70 in lysine K542 by SIRT6 disrupts its interaction with Bax, which finally resulted in Bax translocation. In the current study for the first time we showed that EGCG treatment induced dissociation of c‐FLIP/Ku70 complex.^45^ To the best of our knowledge, no specific report has described the potential of EGCG to induce apoptosis through dissociation of c‐FLIP/Ku70 complex in GC cells.

We addressed this concern in our study by using EGCG at a dose of 100 μM that has been reported to potentially induce apoptosis in GC cells.^45^, ^46^ Our data confirmed the efficiency of this dose to induce apoptosis by increasing the number of early apoptotic cells (Figure 1A,B). As already mentioned in some studies, EGCG can increase the number of cells in early apoptosis phase of different cancers.^32^ This result is supported by the enhanced expression of *P53* and *P21*, two key‐apoptotic genes, observed in the EGCG‐treated cells as compared to the control group (Figure 1C). Furthermore, we demonstrated that the protein expression of c‐FLIP was reduced after EGCG treatment which was compatible with previous studies (Figure 2A,B,E,F).^47^, ^48^

The translocation of acetylated Ku70 from the cytoplasm to the nucleus after treatment with EGCG has been described in previous studies.^49^, ^50^ According to our IF (Figure 2C,D) and sub‐cellular fractionation analyses (Figure 3A,B), we demonstrated that Ku70 protein was translocated from cytoplasm to the nucleus after treatment with EGCG. Based on these findings, we suggested that treatment with EGCG as an HDACi could induce Ku70 acetylation. In addition, numerous studies showed that inducing apoptosis can cause G2/M arrest in treated cells^51^ and decrease the number of cells in S phase.^44^ In contrast, treatment of MKN‐45 cells with 1 μm MG149 did not significantly change the proportion of the cells in G2/M phase, although a significant reduction at S phase was measured (Figure 1E). In our study, cell cycle assay showed that after treatment with EGCG, the population of cells in S phase was drastically decreased and cells were arrested in G2/M phase.

Our IF and Co‐IP data demonstrated that Ku70 contributes to the inhibition of apoptosis by establishing a complex with c‐FLIP in the cytoplasm of MKN‐45 cells. The increased rate of apoptosis following the dissociation of the c‐FLIP/Ku70 complex in MKN‐45 cells consequently to EGCG treatment is an additional evidence that confirms the role of established c‐FLIP/Ku70 in apoptosis inhibition. Based on IF and Sub‐cellular fractionation results, we speculate that following the dissociation of this complex, Ku70 will return to the nucleus where it exerts its normal function as a nuclear DNA repair factor and the cytoplasmic level of c‐FLIP will be decreased.

Altogether, our data showed that EGCG, a natural HDAC inhibitor, dissociates c‐FLIP/Ku70 complex in MKN‐45 GC cells and this is accompanied by the induction of extrinsic apoptosis pathway (summarized in graphical abstract). Our results suggested that EGCG could be an alternate component to the conventional HDAC inhibitors in order to induce apoptosis in GC cells. Furthermore, our data warrant more research on the cellular and molecular mechanisms involved in the apoptosis induction through extrinsic pathway by dissociating this complex to figure out whether combination of EGCG with other cancer therapy protocols can result in better therapeutic outcome.

## AUTHOR CONTRIBUTIONS

**Mahtab Shahriari Felordi:** Investigation (lead); methodology (equal); writing – original draft (equal). **Mehdi Alikhani:** Data curation (supporting); formal analysis (supporting); investigation (supporting). **Zahra Farzaneh:** Data curation (supporting); investigation (supporting); methodology (supporting). **Mahmoud Alipour Choshali:** Data curation (supporting); formal analysis (equal); investigation (supporting); methodology (supporting). **Marzieh Ebrahimi:** Data curation (equal); formal analysis (equal); writing – review and editing (equal). **Hamidreza Aboulkheyr Es:** Data curation (equal); formal analysis (equal); writing – review and editing (equal). **Abbas Piryaei:** Data curation (equal); formal analysis (equal); writing – review and editing (equal). **Mustapha Najimi:** Conceptualization (equal); resources (supporting); writing – review and editing (equal). **Massoud Vosough:** Conceptualization (equal); funding acquisition (lead); investigation (lead); resources (equal); supervision (equal); writing – review and editing (equal).

## FUNDING INFORMATION

This study was funded by grants from Bahar Tashkhis Teb Co, (R.98005) and Royan Institute (R1398‐0335).

## CONFLICT OF INTEREST STATEMENT

The authors have no conflict of interest to declare.

## Supporting information


Figure S1:

## Data Availability

Data available on request from the authors.
