# Supplementary material for: (‐)‐Epigallocatechin‐3‐gallate induced apoptosis by dissociation of c‐FLIP/Ku70 complex in gastric cancer cells
Source: J Cell Mol Med. 2023 Aug 3;27(17):2572–82. doi: 10.1111/jcmm.17873 (PMC10468655; doi:10.1111/jcmm.17873)
Supplement: Supplementary file 1 — Figure S1: [file JCMM-27-2572-s001.docx]

**Supplementary Figure 1**


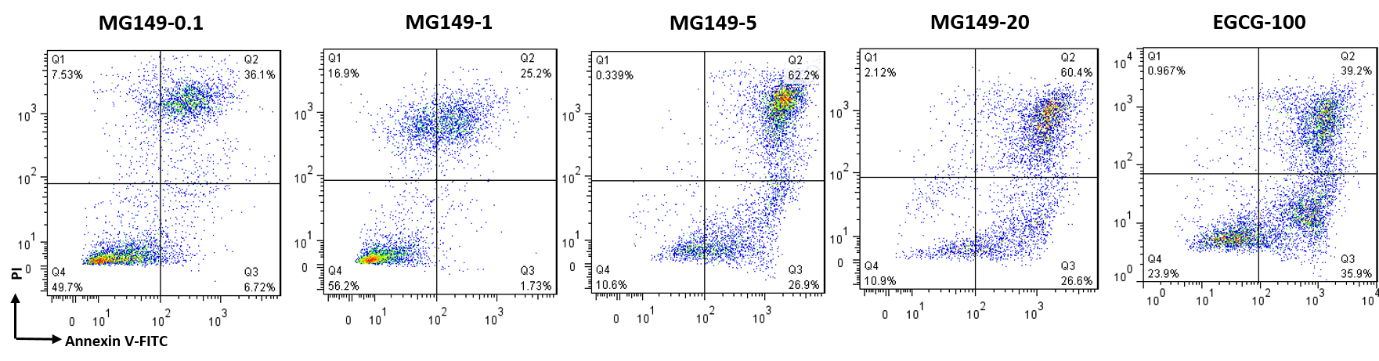


**MG149 dose escalation effect on the viability of MKN-45 cells.** Candidate doses (0.1, 1, 5, 20 and 100 μM) of MG149 were selected and their effects evaluated on apoptosis after 24h treatment, by using flow cytometry. While cell death was more pronounced starting from 5 µM, both 0.1 and 1 µM MG149 low used doses, showed the lesser effects. Based on these data and already other published studies, the dose of 1 μM was selected as the appropriate one to be used for the treatment of MKN-45 cells in this study.
